# Comparative Performance of Machine Learning Models Using Food Intake Frequency Versus Vegetable Intake Data to Predict Problematic Mealtime Behaviour in Japanese Preschool Children

**DOI:** 10.34763/jmotherandchild.20263001.d-25-00036

**Published:** 2026-06-08

**Authors:** Naoki Sakane, Yaeko Kawaguchi, Junichiro Somei, Akiko Suganuma, Masayuki Domichi

**Affiliations:** Division of Preventive Medicine, Clinical Research Institute, National Hospital Organisation NHO Kyoto Medical Centre, Kyoto, Japan

**Keywords:** machine learning, food, vegetable, mealtime behaviour, preschool children, machine performance

## Abstract

**Background:**

Selective eating often leads to nutritional imbalance and mealtime stress, making early identification essential.

**Objective:**

This study aimed to compare the predictive performance of machine learning (ML) models using two types of input data—namely, vegetable intake and food frequency questionnaire (FFQ) responses—to identify children with selective eating behaviour (cutoff score ≥1.59).

**Material and methods:**

We analysed a cross-sectional dataset of 283 children aged 3–6 years using up to 8 predictors selected from 15 food-frequency items or 15 designated vegetables, along with age and sex. Selective eating behaviour was defined as a selective eating score of ≥ 1.59. Eleven ML algorithms were trained and evaluated, resulting in 11,253 feature–model combinations. Model performance was assessed using 5-fold cross-validation based on accuracy, precision, recall, F1 score, and ROC-AUC. Permutation importance analysis was conducted to identify key predictive features.

**Results:**

Among the 11 ML algorithms tested, vegetable intake-based L1 logistic regression showed the best performance (ROC-AUC = 0.717, accuracy = 0.647, precision = 0.722, recall = 0.659, F1 = 0.808). Permutation importance further identified tomato, green onion, and taro as key contributors in the vegetable intake-based L1 logistic regression model. In contrast, the FFQ-based Naïve Bayes model showed relatively good discrimination (ROC-AUC = 0.619) despite moderate recall.

**Conclusion:**

Vegetable intake-based ML models were more effective than those using FFQ data in identifying children with selective eating behaviour. Detailed dietary assessment aids early detection.

## Introduction

Picky eating (selective eating) is a common concern in early childhood and is characterised by limited acceptance of food varieties and frequent refusal to specific food items [1,2,3]. Although often regarded as a developmental phase, persistent selective eating can negatively affect nutritional status, growth, and psychosocial functioning [4,5]. Thus, identifying children at risk for problematic mealtime behaviour is crucial for early intervention [6,7].

Traditional dietary assessments, such as food frequency questionnaires (FFQs), provide a broad overview of food intake; however, they may not fully capture the nuanced, item-specific behaviours associated with food selectivity [8,9]. In contrast, preference-based questionnaires assessing children's responses to individual foods provide more detailed insights into eating behaviour patterns [10,11]. However, these data are often highly dimensional and complex, presenting challenges for conventional statistical analyses.

Recent advances in machine learning (ML) offer promising tools for classifying behavioural patterns and extracting predictive features from multidimensional datasets [12,13]. ML models, particularly tree-based ensemble methods such as XGBoost, have shown utility for predicting health-related behaviours [14] but are underutilised in research on early childhood nutrition.

The present study aimed to compare the predictive performance of various ML algorithms using two types of inputs—namely, (i) FFQ-based food intake frequency data and (ii) item-level vegetable intake data. This study sought to determine the data source and model combination that could most effectively identify children with selective eating behaviour, defined using a validated cutoff. Additionally, this study explored the relative importance of specific food items and demographic features to inform future screening and intervention strategies.

## Material and methods

### Study Design and Participants

This study adhered to the principles outlined in the Declaration of Helsinki and was approved by the Ethics Committee of the National Hospital Organization Kyoto Medical Center (approval number: 24-071). Informed consent was obtained from all caregivers before participation. This study was registered in the UMIN Clinical Trials Registry (registration number: UMIN000057312). All participants provided written informed consent after receiving a thorough explanation of the study protocol.

Children aged 3–6 years were included in the study. The exclusion criteria were as follows: (i) individuals aged <3 years or >7 years and (ii) individuals whose parents or caregivers were unable to read Japanese.

### Measurement

Demographic data, including age and sex, were collected. Dietary assessments were based on the parents' or caregivers' reports. Children's dietary patterns were evaluated using the following eight food groups as primary predictor variables: grains (rice, bread, and noodles), fish, meat, eggs, soybeans/soy products, vegetables, fruits, and milk. Processed food intake was assessed using four items: sweetened beverages, confectionery, instant noodles, and fast foods. The survey inquired how frequently the children consumed foods from each group, with response options ranging from “≥2 times/day” to “rarely or less than once per week” [15]. The frequency of vegetable intake was calculated based on 15 vegetables identified by the Ministry of Agriculture, Forestry, and Fisheries, Japan. Dark green and yellow vegetables include tomatoes, carrots, green peppers, spinach, and broccoli. Other vegetables included cabbage, cucumbers, daikon radishes, Chinese cabbage, onions, eggplants, scallions, and lettuce.

Problematic mealtime behaviour, specifically selective eating, was assessed using the following 11 items from the “Selective Eating” subscale of the Autism Spectrum Disorder-Mealtime Behaviour Questionnaire [16,17,18]:

(i) The child only eats a few types of foods.
(ii) The child only eats certain foods/ingredients or products from certain manufacturers.
(iii) The child does not eat food with a strong smell.
(iv) The child does not eat some foods due to their appearance.
(v) The child does not want the tastes to be blended.
(vi) The child does not eat some foods because of colour.
(vii) Places to eat out are limited/restricted.
(viii) The child does not eat food that he or she has never eaten.
(ix) The child does not eat school meals.
(x) The child eats a narrow variety of foods.
(xi) The child does not eat food.

Parents or caregivers rated how frequently their children engaged in each behaviour over the past week using a five-point Likert scale, with “1” indicating that the behaviour was never observed and with “5” denoting that the behaviour was always observed. The items addressed behaviours such as eating only a limited variety of foods; avoiding foods with strong smells; rejecting foods based on appearance, colour, or changes in shape; and refusing unfamiliar or school-provided meals. Based on our previous validation study (Nakaoka et al., 2024) [18], selective eating behaviour was defined using a mean cutoff score of 1.59, which demonstrated the optimal discriminative performance (AUC = 0.831 [95% CI: 0.782–0.881], sensitivity = 0.695, specificity = 0.820). This threshold was determined using receiver operating characteristic (ROC) analysis to maximise the balance between sensitivity and specificity.

## Feature Selection

Among the candidate variables, a subset of 10 features (age, sex, and eight food or vegetable items) was selected for comprehensive analysis. These features were chosen in descending order of magnitude of difference between the two groups. Parameter optimisation was conducted using GridSearchCV within a 5-fold stratified cross-validation framework to ensure robust model selection.

### Data Preprocessing

The responses were cleaned, normalised, and numerically coded. Participants with missing or incomplete responses were excluded from the analysis. The dataset was then split into training and test sets (70/30 split) using stratified sampling to maintain class balance, and model performance was evaluated using 5-fold cross-validation to ensure robustness and minimise sampling bias. A total of 11,253 feature–model combinations were trained and evaluated across all algorithms. To address class imbalance, the synthetic minority oversampling technique (SMOTE) was applied.

### ML Models

Eleven ML models were tested for their ability to predict selective eating behaviour: Random Forest, Support Vector Machine (SVM), Naive Bayes, Neural Network, k-Nearest Neighbours (k-NN), Extreme Gradient Boosting (XGBoost), Stochastic Gradient Descent Classifier (SGDClassifier), XGBoost limited tree depth (XGB_limitdepth), L1LogisticRegression, Logistic Regression, and Light Gradient Boosting Machine (LightGBM). Each model was trained using two sets of features—namely, (i) FFQ data and (ii) vegetable intake data. Demographic variables (age and sex) were included in some models for comparison. Accuracy, precision, recall, F1 score, and ROC-AUC, all derived from the confusion matrix, were computed to assess the predictive performance of the classification models. Accuracy, which measures the overall proportion of correct predictions, was calculated as (TP + TN) / (TP + TN + FP + FN). Precision, which refers to the proportion of predicted positive cases that are positive and reflects the model's ability to avoid false positives, was calculated as TP / (TP + FP). Recall (sensitivity), which is the proportion of actual positive cases correctly identified and indicates the model's ability to detect true positives, was calculated as TP/(TP + FN). F1 score, which is the harmonic mean of precision and recall, was calculated as F1 score = 2 × (precision × recall) / (precision + recall). This metric provides a balanced measure of both false positives and false negatives. The ROC-AUC quantifies the model's ability to discriminate between classes across all threshold levels [19]. These metrics were used to compare and interpret the performances of the five ML models. Feature importance was assessed using permutation importance or built-in feature importance (for tree-based models).

### Bias

The cross-sectional design limited the ability to infer causality between food intake and selective eating owing to the lack of temporal information. Self-reported stress measures may have introduced information bias, whereas unmeasured caregiver-related factors may have served as residual confounders. Selection bias may also be present, and the relatively small sample size relative to the number of predictors may increase the risk of model overfitting.

### Sample Size

A total sample size of 200 participants was estimated, assuming 10 variables and applying the criterion of EPV ≥20 [20,21].

### Statistical Analysis

ML analyses were conducted using Python (version 3.13.7; Python Software Foundation). The following libraries were utilised: scikit-learn (version 1.6.1) for data preprocessing, feature scaling, model training, and performance evaluation; imbalanced-learn (version 0.13.0) for implementing the Synthetic Minority Over-sampling Technique (SMOTE) to address class imbalance; numpy and pandas for data manipulation and numerical computation; matplotlib and seaborn for visualising model performance; and scipy for statistical testing and performance metrics. Model performance was assessed using 10-fold stratified cross-validation to ensure generalizability, and GridSearchCV was applied for hyperparameter tuning to optimise predictive performance across algorithms. Data were presented as mean ± standard deviation or as percentage (%). The participants were classified into either the control group or the selective eating group. Between-group comparisons were conducted using the independent t-test for continuous variables and Fisher's exact test for categorical variables.

## Results

### Participants and Dietary Assessment

A total of 449 children were screened for eligibility. Of these, 166 were excluded because they were younger than 3 years, older than 6 years, or had missing data. Consequently, 283 children (median age [25th, 75th percentile]: 4.6 ± 1.0 years; 58.3% male) met the inclusion criteria and were included in the final analysis. Of the 283 children included in the analysis, 162 (57.2%) exhibited selective eating behaviour, and 121 (42.8%) were classified as controls. There were no significant differences between the control and selective eating groups in age (4.6 ± 1.0 vs. 4.6 ± 0.9 years, P = 0.562) or in the proportion of boys (59.5% vs. 57.4%, P = 0.808). Table 1 presents the mean weekly intake frequency of 15 general food categories and 15 specific vegetables in the control (*n* = 121) and selective eating (*n* = 162) groups. Significant group differences in overall vegetable intake (*P* < 0.001), fruit intake (*P* = 0.001), sweetened beverage consumption (*P* < 0.001), and fast food consumption (*P* = 0.015) were observed, with lower consumption among children with selective eating behaviour. Among the designated vegetables, children with selective eating behaviour reported significantly lower weekly intake of cabbage, cucumbers, taroes, daikon radishes, tomatoes, eggplants, carrots, green onions, Chinese cabbage, green peppers, lettuce, onions, potatoes, spinach, and broccoli (all *P* < 0.05).

**Table 1.** Food and vegetable frequency per week.

**Variables**	**Control (*n* = 121)**	**Selective eating (*n* = 162)**	***P* value**

Demographic variables Age, years Male, %	4.6 ± 1.0 59.5%	4.6 ± 0.9 57.4%	0.562 0.808
FFQ			
Rice	12.7 ± 3.1	12.4 ± 3.1	0.403
Bread	5.1 ± 2.9	5.2 ± 2.6	0.887
Noodle	1.8 ±1.1	1.7 ± 1.5	0.963
Fish	2.6 ± 1.6	2.5 ± 2.0	0.677
Meat	5.5 ± 3.2	5.0 ± 2.7	0.158
Eggs	3.6 ± 2.3	3.2 ± 2.6	0.109
Beans/tofu	3.7 ± 2.6	3.1 ± 2.7	0.063
Vegetable	11.2 ± 3.9	9.0 ± 5.0	<0.001^*^
Fruit	5.5 ± 4.0	4.0 ± 3.2	0.001^*^
Dairy products	8.1 ± 4.3	8.1 ± 4.3	0.931
Tea	12.9 ± 3.4	12.7 ± 3.0	0.682
Sweetened beverage	2.1 ± 2.3	3.5 ± 3.3	<0.001^*^
Snack	4.9 ± 2.9	5.3 ± 3.6	0.323
Instant foods	0.4 ± 0.5	0.5 ± 0.6	0.846
Fast foods	0.5 ± 0.4	0.7 ± 0.7	0.015^*^

Designated vegetables			
Cabbage	2.6 ± 1.9	1.7 ± 1.5	<0.001^*^
Cucumber	3.0 ± 2.4	2.2 ± 1.8	0.001^*^
Taro	0.7 ± 0.7	0.3 ± 0.5	<0.001^*^
Daikon radish	1.8 ± 1.5	1.3 ± 1.4	0.002^*^
Tomato	3.9 ± 3.3	2.2 ± 2.5	<0.001^*^
Eggplant	1.4 ± 1.4	0.8 ± 1.1	<0.001^*^
Carrot	4.4 ± 2.7	3.8 ± 2.6	0.033^*^
Green onion	1.9 ± 1.7	1.1 ± 1.4	<0.001^*^
Chinese cabbage	1.6 ± 1.6	1.0 ± 1.2	<0.0001^*^
Green pepper	1.5 ± 1.3	0.9 ± 1.2	<0.001^*^
Lettuce	1.9 ± 2.2	1.0 ± 1.4	<0.001^*^
Onion	4.7 ± 2.7	3.6 ± 2.5	0.001^*^
Potato	3.1 ± 2.1	2.2 ± 1.7	<0.001^*^
Spinach	2.3 ± 1.6	1.6 ± 1.6	<0.001^*^
Broccoli.	3.0 ± 2.0	2.1 ± 2.0	<0.001^*^

Mean±standard deviation (SD) or percentage (%).

* *P* < 0.05.

### FFQ-based ML

**Figure 1.**
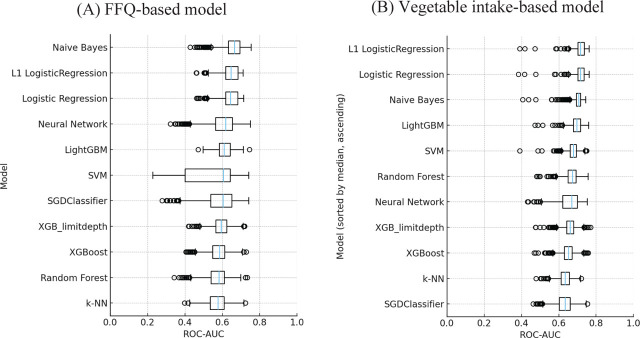
Distribution of ROC–AUC values by machine-learning model. *Note*: Each box represents the interquartile range (IQR) of ROC–AUC values across all configurations for each model, with horizontal lines indicating the median and whiskers extending to 1.5× IQR. The horizontal axis shows ROC–AUC (0–1), and models are arranged from lowest to highest median AUC. Naive Bayes and Neural Network achieved the highest central performance, whereas Random Forest and k-NN showed relatively lower discriminative ability.

**Figure 2.**
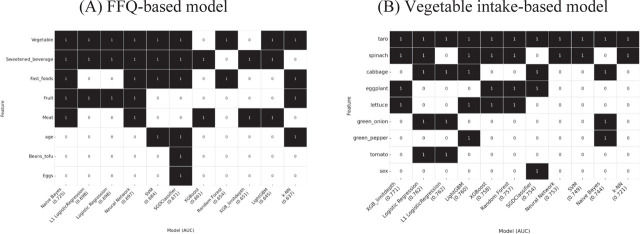
Feature occurrence across best-performing models using (A) Food Frequency Questionnaire (FFQ) categories and (B) individual vegetable intake variables. *Note:* Each cell indicates whether a given feature was selected as important (black = included; white = excluded) in the best-performing model for each algorithm. Vegetable and sweetened beverage intake frequently appeared among FFQ-based models, while taro, spinach, and green onion were consistently selected in vegetable-based models, indicating robust predictive value across algorithms.

**Figure 3.**
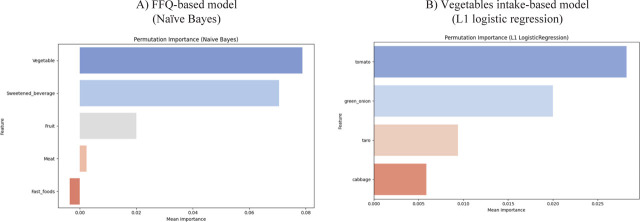
Permutation importance in (A) FFQ-based model (Naïve Bayes) and (B) vegetable intake model (L1 logistic regression). *Note:* Bars indicate the mean decrease in model performance when each variable was permuted, representing its relative contribution to prediction accuracy. In the FFQ-based model (A), vegetable and sweetened beverage intake were the most important, whereas in the vegetable intake-based model (B), tomato, green onion, and taro were key predictors.

Table 2 summarises the mean performance of 11 ML algorithms for predicting selective eating behaviour in preschool children, using up to 8 predictors selected from 15 food-frequency items together with age and sex, evaluated through 5-fold cross-validation. Among all models, L1 Logistic Regression achieved the highest overall performance (ROC-AUC = 0.618, accuracy = 0.585, precision = 0.622, recall = 0.731, F1 = 0.663), followed closely by standard Logistic Regression (ROC-AUC = 0.615, accuracy = 0.582, F1 = 0.658) (Figure 1). The Naïve Bayes model also showed relatively good discrimination (ROC-AUC = 0.619) despite moderate recall. In contrast, tree-based models such as Random Forest, XGBoost, and LightGBM yielded lower ROC-AUC values (~0.51–0.52) and moderate F1 scores (0.58–0.61). Figure 2A illustrates the occurrence of key dietary features across the best-performing ML models using the food-frequency questionnaire (FFQ) dataset. Among all predictors, vegetable intake and sweetened beverage consumption appeared most frequently across high-performing models, indicating their consistent contribution to the prediction of selective eating. Fruit intake was also a common predictor, whereas fast food and meat were less consistently associated. Other variables, such as age, beans/tofu, and eggs, were rarely selected, suggesting limited predictive relevance. Overall, the predominance of vegetable- and beverage-related features highlights the strong influence of healthy versus unhealthy dietary balance in identifying selective eating behaviour among preschool children. Figure 3A presents the permutation importance of major dietary features in the Naïve Bayes model. Among all predictors, vegetable intake had the highest importance, followed by sweetened beverage consumption, both of which contributed strongly to model performance. Fruit intake had a moderate influence, whereas meat and fast food showed minimal effects on prediction accuracy.

**Table 2.** Comparison of machine learning algorithms for predicting selective eating in preschool children using food frequency data (5-fold cross-validation).

**Model**	**ROC-AUC**	**Accuracy**	**Precision**	**Recall**	**F1 Score**
Random Forest	0.522	0.527	0.581	0.611	0.592
SVM	0.543	0.559	0.599	0.722	0.645
Naïve Bayes	0.619	0.578	0.673	0.553	0.578
Neural Network	0.553	0.535	0.584	0.614	0.558
k-NN	0.519	0.527	0.583	0.597	0.585
XGBoost	0.511	0.520	0.575	0.604	0.585
XGBoost (limited depth)	0.521	0.523	0.577	0.612	0.590
SGDClassifier	0.544	0.537	0.595	0.612	0.585
L1 LogisticRegression	0.618	0.585	0.622	0.731	0.663
Logistic Regression	0.615	0.582	0.621	0.718	0.658
LightGBM	0.514	0.527	0.575	0.650	0.605

k-NN, k-Nearest Neighbours; LightGBM, Light Gradient Boosting Machine; ROC-AUC, area under the receiver operating characteristic curve; SGDClassifier, Stochastic Gradient Descent Classifier; SVM, Support Vector Machine; XGBoost, Extreme Gradient Boosting.

### Vegetable Intake-based ML

Table 3 summarises the mean performance of 11 ML algorithms for predicting selective eating behaviour in preschool children, using up to 8 predictors selected from 15 designated vegetable items along with age and sex, evaluated through 5-fold cross-validation. Among all models, L1 Logistic Regression achieved the highest overall performance (ROC-AUC = 0.717, accuracy = 0.647, precision = 0.722, recall = 0.659, F1 = 0.808), followed closely by standard Logistic Regression (ROC-AUC = 0.715, accuracy = 0.646, F1 = 0.798). The Naïve Bayes model also demonstrated strong discriminative ability (ROC-AUC = 0.702, F1 = 0.823), while LightGBM and SVM showed moderate yet consistent performance (ROC-AUC = 0.695 and 0.676, respectively). For the best model, L1 Logistic Regression (implemented via scikit-learn's LogisticRegression), the final parameters were as follows: penalty = ‘l1’; solver = ‘saga’; max_iter = 5000; C = 1; class_weight = None; tol = 1e-4. In contrast, simpler non-linear models such as k-NN and SGDClassifier exhibited relatively lower ROC-AUC values (0.63–0.66). Overall, similar to the food-frequency dataset, linear models again outperformed tree-based and non-linear models, indicating that selective eating behaviour was more accurately captured by linear relationships between vegetable consumption patterns and behavioural outcomes.

**Table 3.** Comparison of machine learning algorithms for predicting selective eating in preschool children using vegetable frequency data (5-fold cross-validation).

**Model**	**ROC-AUC**	**Accuracy**	**Precision**	**Recall**	**F1 Score**
Random Forest	0.668	0.624	0.673	0.674	0.681
SVM	0.676	0.623	0.691	0.653	0.747
Naïve Bayes	0.702	0.638	0.722	0.648	0.823
Neural Network	0.656	0.602	0.633	0.652	0.678
k-NN	0.633	0.603	0.657	0.653	0.673
XGBoost	0.649	0.612	0.659	0.664	0.663
XGBoost (limited depth)	0.660	0.614	0.662	0.666	0.667
SGDClassifier	0.630	0.595	0.638	0.647	0.661
L1 LogisticRegression	0.717	0.647	0.722	0.659	0.808
Logistic Regression	0.715	0.646	0.719	0.661	0.798
LightGBM	0.695	0.639	0.685	0.690	0.691

*Abbreviations:* k-NN, k-Nearest Neighbours; LightGBM, Light Gradient Boosting Machine; ROC-AUC, area under the receiver operating characteristic curve; SGDClassifier, Stochastic Gradient Descent Classifier; SVM, Support Vector Machine; XGBoost, Extreme Gradient Boosting.

Figure 2B shows the occurrence of key vegetable features across the best-performing ML models using the vegetable frequency dataset. Among all predictors, carrot, tomato, and taro were most consistently selected across high-performing models, suggesting a strong association with selective eating behaviour. Chinese cabbage, spinach, and cabbage were also frequently identified, suggesting their potential to contribute to children's dietary diversity. In contrast, daikon radish, green pepper, and sex appeared infrequently, implying limited predictive relevance. In the vegetable-specific L1 logistic regression model, tomato, green onion, and taro contributed most to predictive performance (Figure 3B).

## Discussion

In this study, linear models, especially L1 and standard Logistic Regression, outperformed non-linear models in predicting selective eating. The vegetable frequency data showed higher predictive accuracy than food frequency data, suggesting that vegetable intake patterns are stronger indicators of selective eating behaviour in preschool children.

### FFQ-based ML

In this study, ML models were applied to food-frequency questionnaire (FFQ) data to predict selective eating in preschool children. Although linear models such as L1 and standard Logistic Regression performed slightly better than non-linear and ensemble models, overall predictive accuracy was modest (best ROC-AUC = 0.618). The limited performance suggests that conventional FFQ data may not adequately capture the complexity of selective eating behavior.

Feature analyses identified vegetable intake and sweetened beverage consumption as key predictors, indicating that selective eating reflects an imbalance between healthy and unhealthy dietary patterns. However, the narrow range of influential variables and the low overall discrimination highlight the constraints of FFQ-based modelling.

In summary, while ML can extract meaningful dietary signals from FFQ data, its predictive ability remains limited by the coarse nature of FFQ measurements. Combining FFQs with more detailed, objective dietary assessments—such as digital food logs or behavioural indicators—may enhance the accuracy and interpretability of ML-based models for identifying selective eating in young children.

### Vegetable Intake-based ML

In this study, ML models were applied to vegetable intake frequency data to predict selective eating behaviour in preschool children. Compared with the food-frequency dataset, the vegetable-based models showed superior predictive performance, with the best model (L1 Logistic Regression) achieving an ROC-AUC of 0.717, indicating a more robust relationship between vegetable consumption patterns and selective eating tendencies. Linear models, particularly L1 and standard Logistic Regression, again outperformed non-linear and ensemble models, suggesting that selective eating behaviour can be effectively captured through linear associations among vegetable intake variables.

Feature occurrence analysis revealed that carrot, tomato, and taro were the most frequently selected predictors across top-performing models, highlighting their consistent association with selective eating. Additionally, Chinese cabbage, spinach, and cabbage were often identified, suggesting that variety in vegetable consumption may reflect broader dietary diversity.

Permutation importance analysis using the L1 Logistic Regression model (Figure 3B) further demonstrated that green pepper and cabbage were particularly influential variables, indicating that these vegetables may serve as sensitive indicators of selective eating behaviour. Notably, the opposing tendencies—such as avoidance of bitter-tasting vegetables (e.g., green pepper) and preference for familiar or mild-tasting ones (e.g., carrot)—may represent key behavioural dimensions underlying children's selective eating patterns.

Overall, these findings suggest that vegetable intake frequency data provide more informative and discriminative features for predicting selective eating than general FFQ data. ML approaches, particularly linear models, can effectively identify specific vegetable-related predictors that capture both avoidance and preference tendencies. Such insights may help inform tailored nutritional interventions to increase vegetable acceptance and dietary variety among preschool children.

In the vegetable intake–based L1 logistic regression model, tomato, green onion, and taro contributed most to predictive performance, indicating that both overall and specific vegetable consumption patterns are closely associated with selective eating behaviour. Tomatoes emerged as a key predictor of selective eating in approximately half of the ML models. Despite the familiarity with tomatoes as vegetables, their strong sensory attributes, such as acidity, distinctive odour, and variable texture, may elicit aversive responses, particularly among children with sensory sensitivities or autism spectrum disorder (ASD) traits who often prefer foods with consistent characteristics [22,23]. The common rejection of raw tomatoes, along with the acceptance of ketchup, illustrates item-specific selectivity. These findings suggest that children's responses to tomatoes may serve as behavioural markers for identifying selective eating patterns.

Evidence from systematic studies suggests that early exposure to various vegetables promotes greater acceptance later in life because early dietary habits often persist into adulthood [24]. Among different approaches, taste exposure interventions have demonstrated the greatest effectiveness in increasing vegetable intake during early childhood [25]. Supporting this, a test-meal study by Cooke et al. (2006) involving 4- to 5-year-old children in the UK revealed that higher food neophobia scores, as measured by the six-item Child Food Neophobia Scale, were significantly associated with lower intake of grapes, tomatoes/carrots, chicken, and cheese [26].

Green peppers were identified as a key predictor of selective eating behaviour in the LightGBM and Naïve Bayes models. The strong sensory characteristics of green peppers, particularly their bitterness and pungency, elicit aversion, especially in children with heightened taste sensitivity or sensory overresponsivity, such as those with ASD. Variability in texture and lingering skin may also contribute to discomfort. In Japanese culture, green peppers are commonly served yet often disliked, reinforcing their status as salient markers of picky eating. These findings highlight the utility of item-specific food responses in detecting selective eating. The study conducted by Dazeley et al. (2015), which involved 92 toddlers aged 12–36 months, demonstrated that daily exposure to the sensory properties (sight, touch, smell, and sound) of unfamiliar fruits and vegetables, such as green peppers, over four weeks increased children's willingness to touch and taste these foods during mealtimes [27].

### Future Perspective

Future studies should integrate multidimensional data sources to improve the prediction and understanding of selective eating. These may include food preference and sensory response data (e.g., taste sensitivity, texture aversion), psychosocial variables (e.g., emotional reactivity, parental feeding practices), contextual and environmental factors (e.g., meal settings, peer influence), and longitudinal tracking to capture behavioural changes over time. Additionally, externally validating models for larger, more diverse populations is essential to enhance generalizability. Interpretable, high-performing models may eventually be applied in clinical, educational, or public health settings to screen children at risk and support early tailored interventions for improving dietary habits and mealtime behaviour. The developed ML models can serve as screening tools to identify preschool children at risk of selective eating using simple information such as age, sex, and food intake frequency. In clinical settings, dietitians and paediatricians could apply these models during routine checkups to guide early dietary interventions. In community or school programs, the models could be integrated into digital questionnaires to support early detection and targeted nutrition education. The interpretable nature of logistic regression models further enhances their feasibility for real-world use.

### Strengths and Limitations

First, a key strength of this study is its use of an evidence-based cutoff score (≥1.59) to define selective eating behaviour, enabling clinically meaningful classification and outcome prediction. Second, we conducted a comprehensive comparison of multiple ML algorithms using robust performance metrics, including accuracy, F1 score, and ROC-AUC. Class imbalance was appropriately addressed using the Synthetic Minority Over-sampling Technique (SMOTE), and model performance was evaluated through 5-fold stratified cross-validation, enhancing generalizability and reproducibility. This study applied a comprehensive ML framework using multiple algorithms in Python, allowing for systematic comparison of predictive accuracy across models. This facilitated the identification of optimal models for detecting problematic eating behaviours. Third, the inclusion of feature importance analysis provided practical insights by identifying specific vegetables, such as tomatoes and taros, as significant predictors of selective eating tendencies. Finally, the study emphasises the utility of food-preference data over food-intake frequency, demonstrating that item-level responses better capture the behavioural characteristics associated with selective eating.

However, this study has several limitations that must be acknowledged. First, the sample size was modest, which may restrict the generalizability of the findings to a broader population. However, the final sample size (n = 283) exceeded the estimated requirement (n = 200), ensuring sufficient statistical power and robust model training. Therefore, larger and more diverse datasets are required to validate our results. Second, the cross-sectional design limited the ability to infer causality between the predictors of selective eating behaviour. Longitudinal studies are beneficial for examining behavioural trajectories over time. Third, important factors such as sensory sensitivity, parental feeding practices, and emotional characteristics were not included in the model, potentially reducing the explanatory power. Fourth, while the selective eating score was used to differentiate between children with and without ASD [16,17,18], future research should consider employing or comparing alternative instruments specifically developed for neurotypical children—such as the Children's Food Neophobia Scale (CFNS) [28]—to further validate and extend the present findings. Finally, dietary data were obtained through parent-reported questionnaires, which may be subject to reporting bias: parents tend to overestimate intake of vegetables and other healthy foods while underreporting the frequency of sweets, fast food, and snacks. However, because the same validated questionnaire was used for all participants and responses were collected, the potential bias is unlikely to have systematically influenced group comparisons.

## Conclusion

This study found that vegetable intake-based ML models outperformed those based on FFQ data in predicting selective eating. The L1 Logistic Regression model exhibited the best performance (Figure 3). Key predictors were specific vegetables (e.g., tomatoes, carrots, and green peppers), whereas demographic factors had minimal impact. These results suggest that selective eating is more strongly driven by specific food aversion than by overall food intake patterns or demographic traits.
